# Life-Threatening Acidosis With Metformin and Dapagliflozin Combination Therapy: A Case Report

**DOI:** 10.7759/cureus.35497

**Published:** 2023-02-26

**Authors:** Sushan Gupta, Avani Mohta, Shodunke Temitope

**Affiliations:** 1 Internal Medicine, Carle Foundation Hospital, Champaign, USA; 2 Nephrology and Critical Care, Carle Foundation Hospital, Champaign, USA

**Keywords:** metformin, life-threatening acidosis, dapagliflozin, lactic acidosis, euglycemic acidosis

## Abstract

Euglycemic keto-acidosis is a known complication of dapagliflozin. However, acidosis can be life-threatening when dapagliflozin is used as a combination therapy with metformin. Our patient was a 64-year-old male, with a history of well-controlled type 2 diabetes mellitus on metformin and dapagliflozin, admitted with vomiting and diarrhea for several days. On presentation, the patient was hypotensive and severely acidotic (pH < 6.7; bicarbonate <5 mmol/L) with an anion gap of 47. Other labs included elevated lactate (19.48 mmol/L), creatinine of 10.39 mg/dL, and elevated beta-hydroxybutyrate levels. The patient was intubated and started on dual vasopressors, insulin drip, and i.v. hydration. Due to worsening acidosis, bicarbonate drip and, subsequently, continuous dialysis was started. The patient's acidosis normalized after two days of dialysis, and he was extubated by day three and discharged by day seven. Dapagliflozin leads to keto-acidosis due to increased hepatic ketogenesis and adipose tissue lipolysis. It also promotes natriuresis, glycosuria, and free water loss. Recurrent vomiting and poor oral intake with concomitant lactic acidosis with metformin can lead to life-threatening acidosis. Clinicians should remain cognizant of the possibility of severe acidosis with the combination therapy of dapagliflozin and metformin in severe dehydration. Adequate hydration may prevent this life-threatening complication.

## Introduction

Dapagliflozin is a potent sodium-glucose co-transporter-2 inhibitor (SGLT2i) and is currently recommended as an adjunct in type 2 diabetes mellitus for superior glycemic control and as a pillar of guideline-directed medical therapy for patients with heart failure. SGLT-2 receptors are part of the sodium-glucose transporter family found exclusively in the kidney's proximal tubules. SGLT2i binds to these receptors and prevents the reabsorption of sodium and glucose, leading to glucosuria, natriuresis, and associated osmotic diuresis [1]. Euglycemic keto-acidosis is a well-established life-threatening complication of SGLT2i due to increased ketogenesis, especially with poor oral intake and dehydration [2]. With the increased use of SGLT-2i as a combination therapy with metformin, acidosis can be potentially much more severe in these settings [1]. We present a case of severe euglycemic ketoacidosis and lactic acidosis secondary to poor oral intake and dehydration requiring dialysis in a diabetic patient on chronic dapagliflozin and metformin therapy.

## Case presentation

A 64-year-old male patient with a past medical history significant for type 2 diabetes mellitus and hypertension was admitted with the chief complaints of vomiting and diarrhea for several days, unable to tolerate an oral diet. The patient's medications included dapagliflozin 5 mg daily, metformin 1000 mg twice daily, and glimepiride 4 mg twice daily. Six months ago, the patient had a normal complete blood count and basic metabolic panel, with a serum creatinine of 1.29 mg/dL and an estimated glomerular filtration rate (GFR) of around 56 mL/min.

The patient initially presented to an outside hospital, where labs were consistent with severe metabolic acidosis with a pH of 7.04, bicarbonate of 9 mmol/L, lactic acid of 7.8 mmol/L, and base-excess of 23.4 mmol/L. In addition, the patient had a creatinine of 10.5 mg/dL (reference range: 0.70-1.30 mg/dL) and a blood urea nitrogen (BUN) of 77 mg/dL (reference range: 7-25 mg/dL). The patient was transferred to our hospital for further care.

On arrival, the patient was afebrile and hypotensive, with systolic blood pressure in the 80s. Other vital signs included a heart rate of 110-120's beats/minute, saturating 93-94% on room air. CT chest/abdomen/pelvis done on presentation was unrevealing. Arterial blood gas (ABG) in the emergency department showed severe acidosis, pH < 6.7, arterial partial pressure of carbon dioxide (paCO_2_) 15.2 mmHg, and arterial partial pressure of oxygen (PaO_2_) 141 mmHg. Lactates were 19.48 mmol/L on presentation (Table 1).

**Table 1:** Arterial blood gas at the time of admission PaCO_2_ - arterial partial pressure of carbon dioxide; PaO_2_ - arterial partial pressure of oxygen; tHb - total hemoglobin; O_2_Hb - percent hemoglobin oxygen saturation

Analyte	Value
pH	<6.7
PaCO_2_ (mmHg)	15.2
PaO_2_ (mmHg)	141
Lactate (mmol/L)	16.39
tHb (g/dL)	15.4
O_2_Hb (%)	95.3

The basic metabolic panel showed elevated kidney functions (blood urea nitrogen (BUN) - 85 mg/dL creatinine 10.39 mg/dL), bicarbonate of <5 mmol/L, and normal glucose levels (149 mg/dL). CBC showed leukocytosis (WBC - 21.43 10^3^/uL) and normal hemoglobin (14.4 gm/dL). Other electrolytes included sodium 148mg/dL, potassium 6.2 mmol/L, and chloride 101 mmol/L, with an anion gap of 47 (Table 2).

**Table 2:** Basic metabolic panel at the time of admission

Laboratory test	Result value		Reference range
Calcium (mg/dL)	9.7		8.9 - 10.6
Glucose (mg/dL)	149		74 - 100
Blood Urea Nitrogen (mg/dL)	85		8 - 26
Creatinine (mg/dL)	10.39		0.55 - 1.30
Sodium (mmol/L)	148		136 - 145
Potassium (mmol/L)	6.2		3.5 - 5.1
Chloride (mmol/L)	101		98 - 107
Bicarbonate (mmol/L)	<5		22.0 - 29.0

Beta-hydroxybutyrate levels and glycated hemoglobin (HbA1c) were 7.4 and 7, respectively, on presentation. The patient received 2 liters of normal saline bolus and 150 meq IV sodium bicarbonate before transfer to the ICU. Blood and urine cultures were sent, and the patient was empirically started on i.v. antibiotics.

In the ICU, the diabetic ketoacidosis protocol was started with insulin infusion and intravenous hydration. A bicarbonate drip was also initiated due to severe acidosis. Due to significant respiratory distress, the patient required bilevel-positive airway pressure (BiPAP); however, he was intubated later during the day due to the increased work of breathing and altered mental status. The patient required vasopressor support post-intubation, including both norepinephrine and vasopressin. Due to progressive acidosis, continuous renal replacement therapy was initiated later on the same day of admission.

Patients' acidosis progressively improved, and patients' pH and bicarbonate levels normalized after two days of dialysis. The patient was switched to long-acting Lantus insulin 10U subcutaneously. He was extubated on day three and was off vasopressor support by day four. The blood cultures drawn at the hospital presentation were negative, and antibiotics were discontinued. The patient was shifted to the medical floor on day four. He remained stable throughout the hospital stay and was discharged by day seven on long-acting insulin, Levemir 10 units daily.

## Discussion

Combination therapy of SGLT2i and metformin has been shown to improve HbA1c by at least 0.3-0.5% in recent trials [3]. They are recommended as a combination first-line therapy in patients with established cardiovascular risk [4]. Both medications are individually associated with rare but significant life-threatening acidotic complications; however, studies on whether combination therapy increases the risk of acidosis in clinical practice are lacking. Our case is unusual due to the severity of acidosis requiring dialysis, and mechanical ventilation, in a patient on chronic dapagliflozin and metformin therapy.

Dapagliflozin binds to the sodium-glucose transporter receptors in the proximal convoluted tubules preventing the reabsorption of glucose and sodium, leading to natriuresis, glycosuria, and associated free water loss. In the setting of ongoing dehydration secondary to recurrent vomiting and poor oral intake, as seen in our patient, there is significant volume depletion and associated lactic acidosis and kidney injury. Decreased renal function affects metformin clearance, and in high concentration, it affects hepatic mitochondrial respiration and gluconeogenesis in the liver, preventing lactate utilization and adding to ongoing lactic acidosis [5].

In addition, SGLT2i leads to keto-acidosis due to an increased rate of hepatic ketogenesis and adipose tissue lipolysis by increasing plasma catecholamine and corticosterone levels secondary to volume depletion [6]. As they also reduce blood glucose levels, there is a decrease in the secretion of endogenous insulin, and pancreatic alpha-cells are stimulated to release glucagon, further promoting hepatic ketogenesis [7]. All these mechanisms are compounded in the setting of ongoing volume loss, as seen in our patient with vomiting, diarrhea, and poor oral intake. SGLT2i is associated with an almost 2.5 times higher risk of ketoacidosis than other diabetic medications; however, among the SGLT-2 class of drugs, dapagliflozin has the least association with euglycemic keto-acidosis [8]. 

Identifying euglycemic ketoacidosis is challenging as patients present with normoglycemia due to the glucosuric action of SGLT2i. Low serum bicarbonate levels and acidosis on arterial blood gas analysis are often needed to confirm the diagnosis. Initial treatment of euglycemic ketoacidosis with lactic acidosis is focused on fluid resuscitation, with insulin infusion, until the acidosis resolves and the anion gap normalizes. In contrast to diabetic ketoacidosis, dextrose-containing fluids are often the initial choice in euglycemic ketoacidosis to avoid hypoglycemia and allow for faster clearance of ketosis. In rare instances like ours, patients may present with severe acidosis with acute kidney injury, needing mechanical ventilation and dialysis. However, once the inciting agent is held and subsequent management with hydration, insulin infusion, and dialysis initiated, metabolic abnormalities rapidly normalize to baseline.

## Conclusions

In conclusion, SGLT2i is associated with euglycemic ketoacidosis. In the setting of severe volume depletion and kidney injury, it worsens metformin-associated lactic acidosis. Clinicians should remain cognizant of the possibility of life-threatening acidosis with combination therapy, especially in patients with poor oral intake and dehydration. Early management with fluids and insulin is generally sufficient; however, severe acidosis may require dialysis.
